# Ecological contexts associated with early childhood curiosity: Neighborhood safety, home and parenting quality, and socioeconomic status

**DOI:** 10.3389/fpsyg.2023.986221

**Published:** 2023-02-28

**Authors:** Prachi E. Shah, Kathy Hirsh-Pasek, Maria Spinelli, Jenny Ozor, Heidi M. Weeks, Harlan McCaffery, Niko Kaciroti

**Affiliations:** ^1^Division of Developmental-Behavioral Pediatrics, Department of Pediatrics, University of Michigan Medical School, Ann Arbor, MI, United States; ^2^Department of Psychiatry, University of Michigan Medical School, Ann Arbor, MI, United States; ^3^Department of Psychology, Temple University, Philadelphia, PA, United States; ^4^Department of Neuroscience, Imaging and Clinical Science, University Gabriele d’Annunzio Chieti-Pescara, Chieti, Italy; ^5^Department of Public Policy and Government Affairs, CareFirst Blue Cross Blue Shield, Washington, DC, United States; ^6^Department of Nutritional Sciences, School of Public Health, University of Michigan, Ann Arbor, MI, United States; ^7^Department of Biostatistics, University of Michigan, Ann Arbor, MI, United States

**Keywords:** curiosity, ecological contexts, neighborhood safety, socioeconomic status, parenting quality, home environment

## Abstract

**Introduction:**

Curiosity is an important social-emotional process underlying early learning. Our previous work found a positive association between higher curiosity and higher academic achievement at kindergarten, with a greater magnitude of benefit for children with socioeconomic disadvantage. Because characteristics of the early caregiving and physical environment impact the processes that underlie early learning, we sought to examine early environmental experiences associated with early childhood curiosity, in hopes of identifying modifiable contexts that may promote its expression.

**Methods:**

Using data from a nationally representative sample of 4,750 children from the United States, this study examined the association of multi-level ecological contexts (i.e., neighborhood safety, parenting quality, home environment, and center-based preschool enrollment) on early childhood curiosity at kindergarten, and tested for moderation by socioeconomic status.

**Results:**

In adjusted, stratified models, children from lower-resourced environments (characterized by the lowest-SES tertile) manifested higher curiosity if they experienced more positive parenting, higher quality home environments, and if they lived in “very safe” neighborhoods.

**Discussion:**

We discuss the ecological contexts (i.e., parenting, home, and neighborhood environments) that are promotive of early childhood curiosity, with an emphasis on the role of the neighborhood safety and the “neighborhood built environment” as important modifiable contexts to foster early childhood curiosity in lower-resourced families.

## Introduction

Curiosity is a fundamental human motivation that influences learning, the acquisition of knowledge, and life fulfillment (Kashdan et al., 2020), and in children, is believed to be a critical social-emotional process underlying academic achievement (Lepper et al., 2005). Curiosity is described as the motivational drive to seek out information in new, uncertain, or complex situations (Loewenstein, 1994; Litman, 2005; Jirout and Klahr, 2012), and in young children, it is often expressed by exploratory behavior (Berlyne, 1954), novelty seeking (Berlyne, 1960), and the joy of learning. Higher curiosity has been associated with adaptive outcomes throughout the lifespan, including better academic and interpersonal outcomes in middle childhood (Maw and Maw, 1975; Lepper et al., 2005), adolescence (Kashdan and Yuen, 2007; Jovanovic and Brdaric, 2012; Froiland et al., 2015), and adulthood (Kashdan and Steger, 2007; Kashdan et al., 2013a). Given the beneficial outcomes associated with curiosity across the life-course, we were interested in examining the environmental contexts associated with higher curiosity in *early childhood*.

In our prior work, using data from a nationally representative sample of 6,200 children from the United States, we examined the association between parent-reported curiosity and kindergarten academic achievement. We found a positive association between higher curiosity and higher academic achievement in reading and math at kindergarten, with a greater magnitude of benefit for children with socioeconomic disadvantage (Shah et al., 2018). Our results demonstrated that while higher curiosity was associated with higher academic achievement in *all children*, low-income children with higher curiosity demonstrated the greatest gains in academic achievement, with the achievement gap between high and low-income children essentially eliminated at high levels of early childhood curiosity. One implication from this work was the possibility that promoting curiosity in young children could be one way to mitigate the achievement gap associated with poverty, although the contexts associated with the promotion of curiosity have been relatively understudied (Hassinger-Das and Hirsh-Pasek, 2018).

Curiosity is a multidimensional construct that is both person-specific (i.e., trait curiosity) and situation-specific (i.e., state curiosity). While *trait curiosity* is related to aspects of personality which are highly heritable and less influenced by context (Steger et al., 2007), *state curiosity* is related to an individual’s interests, whose expression can vary with context and experiences (Black and Deci, 2000; Kashdan and Fincham, 2004; Ainley, 2019). Currently, we have a limited understanding of the contexts which can foster early childhood state curiosity, especially in children from under-resourced environments. Addressing this knowledge gap can lead to targeted interventions to support the expression of early childhood curiosity, which could have implications for early academic achievement.

Ecological theory (Brofenbrenner, 1993) has identified the multilevel contexts and social experiences which influence early child development. These contexts include the quality of experiences in the *proximal* (microsystem) environment (e.g., *early parent-child relationship* (Sroufe, 2005), *home environment* (Crosnoe et al., 2010), and *early educational environment* [Fuller et al., 2017)]; the *distal* (mesosystem) environments (e.g., *the safety of the neighborhood* in which children live [Minh et al., 2017)]; and the *macro contexts* (macrosystem) associated with poverty and socio-economic disadvantage (Brooks-Gunn and Duncan, 1997; Heckman, 2006; Hyde et al., 2020). We theorize that the ecological contexts associated with more optimal *early learning* may also be relevant for the promotion of *early childhood curiosity*.

*Proximal contexts* associated with better school readiness skills include more sensitive early parenting (Frick et al., 2018; Snijders et al., 2020), more stimulating home environments (Rodriguez and Tamis-LeMonda, 2011; Hirsh-Pasek et al., 2015a) and enrollment in center-based preschool (Fuller et al., 2017). Because curiosity is fostered in environments which promote inquiry and align with a child’s individual interests, we hypothesize that more positive parenting (characterized by greater sensitivity and cognitive stimulation), more stimulating home environments, and enrollment in preschool may be similarly promotive of early childhood curiosity.

*Distal and macro* ecological contexts salient to early childhood development relate to the socioeconomic conditions in the child’s neighborhood environment, including poverty and neighborhood safety (Leventhal, 2018). Neighborhoods characterized by poverty, disadvantage, and lower neighborhood safety have been associated with lower academic achievement (Leventhal et al., 2015), more behavior problems (Leventhal and Brooks-Gunn, 2000), and structural differences in brain development contributing to impaired emotion regulation (Hyde et al., 2020), although associations with early childhood curiosity have not been examined. Curiosity is characterized by the drive to seek out new information (Loewenstein, 1994) and the desire to explore (Jirout and Klahr, 2012). Because neighborhoods are a salient ecological context for play, exploration, and learning, characteristics of the neighborhood environment, especially *neighborhood safety*, may have implications for the expression of curiosity by fostering or deterring children’s ability to play and explore.

There is some empirical support linking parents’ perceptions of neighborhood safety and children’s play behavior, especially for under-resourced children. Low-income mothers who rated their neighborhood as unsafe or unpredictable were less likely to allow their children to play outside, citing that survival (in a high-crime neighborhood) was a higher priority than outdoor play (Dias and Whitaker, 2013). Other studies have similarly suggested an association between parents’ perception of neighborhood safety and parents’ promotion of outdoor play, with decreased frequency of outdoor play associated with increased parent concerns about neighborhood safety (Kalish et al., 2010; Galaviz et al., 2016).

Relatedly, in our previous work, we observed an “achievement gap” in low-income children who were rated as having lower parent-reported curiosity (Shah et al., 2018). One potential explanation for these findings is that children from under resourced environments may prioritize safety over exploration, which can contribute to the observed achievement gap compared to their more-curious peers (Hassinger-Das and Hirsh-Pasek, 2018). Neighborhood environments which are safer may contribute to greater exploratory behavior, resulting in higher early childhood curiosity. Critically, if greater neighborhood safety is associated with higher early childhood curiosity, this could lead to the development of interventions to optimize neighborhood environments (e.g., through attention to the quality of the built environment), to promote social-emotional processes related to early learning. This is notable because while some contexts associated with early academic achievement (e.g., genetics) are *relatively* immutable, the expression of early childhood curiosity may be malleable with context, and can vary according to the child’s early experiences in the home, early education and neighborhood environments. Identifying the modifiable contexts associated with higher curiosity can lead to the development of interventions to support the expression of early childhood curiosity, which can potentially foster early academic achievement.

The aims of this study were to examine the proximal and distal ecological contexts of early childhood (i.e., parenting quality, quality of the home environment, enrollment in center-based preschool and neighborhood safety), and associations with curiosity at kindergarten, and to test for moderation by socioeconomic status (SES). We hypothesized that early experiences characterized by more positive parenting, higher quality home environments, enrollment in center-based preschool, or greater neighborhood safety would be associated with higher early childhood curiosity, with potentially magnified effects in children with socioeconomic disadvantage.

## Materials and methods

### Study design and sample

Data were drawn from the Early Childhood Longitudinal Study, Birth Cohort (ECLS-B), a nationally representative, population-based longitudinal study sponsored by the US Department of Education’s National Center for Education Statistics (NCES). The ECLS-B is based on a nationally representative probability sample of children born in the United States in 2001 (inclusive). Data were collected from children and their parents at 9 months, 24-months, preschool and kindergarten timespoints, and included home visits with parent interviews, and direct and indirect child assessments across multiple settings (Snow et al., 2009). Our sample excluded children with congenital and chromosomal abnormalities, and included children born at 22–41 weeks gestation who had parental kindergarten behavioral data from which we could derive a measure of curiosity. Our study utilized data from birth certificate data, 24-months, preschool and kindergarten timespoints, and our final analytic sample included 4,750 children who had curiosity data at kindergarten, data on neighborhood safety, and all predictors and covariates (described below). This study was considered exempt by the Institutional Review Board because it involved the use of a publicly available dataset with de-identified participants who could not be linked to the data. Sample characteristics are described in Table 1.

**TABLE 1:** Maternal/Home and child characteristics.

Maternal/Home characteristics	Mean, *SD* or Weighted%
Age (years)^a^	27.4, *5.3*
**Race/Ethnicity^b^**	
White/Non-Hispanic	59.9%
Black/Non-Hispanic	13.6%
Hispanic	20.8%
Asian	3.1%
Other	2.6%
**Marital status^a^**	
Married	69.2%
Unmarried	30.8%
Socioeconomic status at kindergarten^e^	−0.01, *0.96*
Parenting quality (sensitive, scaffolding, stimulating parenting)^c^	4.4, *1.3*
Quality of home environment^c^	7.3, *2.0*
**Child sex^a^**	
Male	51.7%
Female	48.3%
Gestational age (weeks)^a^	38.5, *2.5*
Center-based preschool experience (yes)^d^	56.4%
24 Month cognitive development (T-score)^c^	50.8, *17.2*
Child age at kindergarten (months)^e^	68.2, *7.8*
Child sustained attention (24-months)^c^	4.5, *1.7*

Wave of data collection: ^a^Birth certificate; ^b^9-months; ^c^24-months; ^d^preschool; ^e^kindergarten.

Source: U.S. Department of Education, National Center for Education Statistics, Early Childhood Longitudinal Study, Birth Cohort. Selected years 2001–2007.

### Measures

#### Outcome

##### Early childhood curiosity

Because the ECLS-B did not have a measure to examine curiosity, we derived a measure of curiosity from an existing assessment of child behavior from the kindergarten timepoint, which included questions from the Preschool and Kindergarten Behavioral Scales Second Edition (PKBS-2). While we were limited by the questions that were available on the parent PKBS-2 questionnaire, we drew from previous theoretical work and behavioral descriptions of curiosity in young children to select question items that most closely aligned with characteristics of curiosity. While there is no single definition of curiosity (Kidd and Hayden, 2015), there are certain behavioral characteristics of curiosity that are widely accepted, including, (1) the thirst for knowledge, and the drive to understand what one does not know (Hall and Smith, 1903; Jirout and Klahr, 2012); (2) an exploratory drive to seek novelty (Berlyne, 1954); (3) an openness to new experiences (Maw and Maw, 1966); and, in young children, (4) innovation in exploratory play (Schulz and Bonawitz, 2007; Cook et al., 2011). Four question items from the PKBS-2 which aligned with these characteristics of curiosity were chosen for our “curiosity factor” (Appendix A). At the kindergarten timepoint, parents were asked to report the frequency of behaviors observed in the previous 3 months on a 5-point Likert scale (1, never to 5, very often). Items were reverse coded as appropriate such that higher scores indicated more positive behaviors. A confirmatory factor analysis (CFA) was conducted to assure reliability and to calculate the appropriate loading values for deriving our curiosity factor. Standardized scoring of the curiosity factor was conducted, and good internal consistency was demonstrated (α = 0.70, *M* = 0.07, SD = 1.2) (Shah et al., 2021).

#### Predictors: Relevant multi-level ecological contexts

##### Neighborhood safety

Neighborhood safety was assessed from parent interviews at 24-months, preschool, and kindergarten timepoint. At the 24-month timepoint parents were asked, “*Do you consider your neighborhood very safe from crime, fairly safe, fairly unsafe or very unsafe?”* At preschool and kindergarten timespoints, parents were asked if they have moved s0e the last timepoint, and if yes, parents were asked again, “*Do you consider your neighborhood very safe from crime, fairly safe, fairly unsafe or very unsafe?”* The description of neighborhood safety from the most recent timepoint was coded as the indicator of “neighborhood safety.” Responses were trichotomized (unsafe (combining fairly unsafe and very unsafe responses); fairly safe (ref); very safe).

##### Parenting quality

Three domains of parenting (Sensitivity, Cognitive Stimulation and Positive Regard) were observed and coded independently during a structured parent-child interaction task (Two-Bags task) at 24-months (NICHD Early Child Care Research Network, 1999; Nord et al., 2006). Both parents and children were instructed to interact for 10 min with two different activities (i.e., pretend play with a set of small dishes and joint book reading). The 10-min parent–child interactions were videotaped and coded. *Parent sensitivity* reflects the degree to which parent interactions are responsive and child centered; *Cognitive Stimulation* reflects a parent’s ability to provide effortful teaching to promote language, perceptual and cognitive skills, while being sensitive to the child’s developmental level; *Positive regard* reflects a parent’s expression of warmth, attentiveness, and attunement. Scores for each domain were rated on a 7-point Likert Scale (1–7) with higher scores demonstrating more positive parenting. Because these three domains of parenting are inter-related, a composite parenting score was calculated as the mean score across the three domains.

##### Quality of the home environment

The quality of the home environment at 24-months was assessed from an abbreviated version of the HOME Inventory (8-items of the HOME-SF) selected for the ECLS-B (Bradley et al., 2001). The HOME Inventory is an instrument designed to measure the quality and amount of stimulation in the home environment available to the child (Snow et al., 2007), and captured characteristics thought to be important for the promotion and expression of early childhood curiosity. The abbreviated HOME-SF included questions about verbal stimulation and child-directed speech (e.g., *parent spoke spontaneously to child*), parent stimulation of child’s play (e.g., *parent provided toys to child*), and safety of the home environment (e.g., *parent kept the child in view*, and *play environment was safe*.) Question items were scored as yes/no, and summed, to generate a cumulative HOME score, with higher scores indicating a more optimal home environment (Range: 1–8).

##### Enrollment in center-based preschool

Because preschool enrollment can vary by family socioeconomic status and neighborhood characteristics (Guyol et al., 2022), and because early educational environments can foster the expression of curiosity (Jirout et al., 2018), we also included a measure from the preschool timepoint regarding whether the child attended center-based preschool (yes/no).

##### Socioeconomic status (SES)

We also included a continuous measure of SES from the kindergarten timepoint in our analyses, which was a composite variable calculated by the ECSL-B which included the following components: father’s education, mother’s education, father’s occupation, mother’s occupation, and household income. Per the ECLS-B codebook, each individual component was converted to a standardized z-score with a mean of 0 and standard deviation of 1, with the SES composite variable computed as an average of the individual measures (Snow et al., 2009) (Range: −2.31–2.09). Covariates: In our analyses, we included relevant sociodemographic variables. Specifically, we controlled for child sex, maternal age, and marital status (married/unmarried), ascertained from birth certificate data; maternal race/ethnicity, ascertained at 9-months, and child age at the kindergarten timepoint. In addition, because lower developmental skills may be related to the expression of early childhood curiosity, we also included a measure of infant development at 24-months from the Bayley Short-Form Research Edition (BSF-R) as a covariate. The BSF-R was adapted from the Second Edition of the Bayley Scales of Infant Development (Bayley, 1993; NICHD Early Child Care Research Network, 2005), and assessed children’s cognitive ability (e.g., language skills, reasoning, problem solving, and memory). Relatedly, because a child’s ability to sustain attention with objects is related to the behavioral expression of curiosity (Kidd and Hayden, 2015), we also included a measure of child sustained attention, coded from observations of the child and parent interaction during the Two Bags Task at 24-months. In this task, child behaviors are coded on a scale of 1 (very low) to 7 (very high) and indicate the degree of child sustained attention and involvement with objects, with higher scores indicating higher child-focused attention (Nord et al., 2006).

### Analysis

**FIGURE 1:**
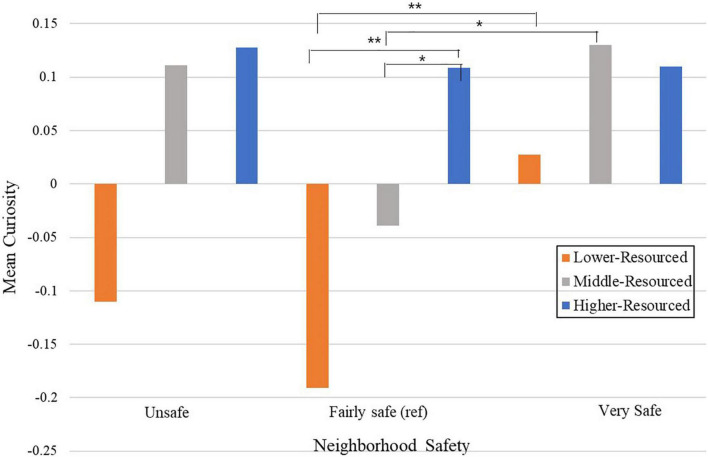
Mean parent-reported curiosity at kindergarten, and neighborhood safety, stratified by lower-, middle-, and higher-resourced environments (SES-tertiles). *p < 0.05; **p < 0.01. Source: U.S. Department of Education, National Center for Education Statistics, Early Childhood Longitudinal Study, Birth Cohort. Selected years 2001–2007.

All analyses were conducted using SAS 9.4 (SAS Institute Inc, 2014) (SAS Institute Inc., Cary, NC, USA). Maternal and child characteristics were examined using descriptive statistics. To test the association between relevant proximal and distal ecological contexts and kindergarten curiosity, we performed multivariable linear regression utilizing the SURVEYREG (SAS) procedure. We included relevant socio-demographic characteristics (i.e., child sex, maternal age, marital status, maternal race/ethnicity, child age at the kindergarten timepoint) and covariates related to early childhood curiosity (i.e., cognitive development and sustained attention at 24-months) to adjust for theoretically justified confounds. For our primary analysis (main effects model), we tested the association between parenting quality, home environmental characteristics, enrollment in center-based preschool (*proximal ecological contexts*), level of neighborhood safety (*distal ecological context*), and SES (*macro context*) with curiosity at kindergarten. We then tested four moderation models, examining whether the association between neighborhood safety and child curiosity was moderated by (1) parenting quality; (2) quality of the home environment (3) enrollment in center-based preschool and (4) SES. In our moderation analyses, we included the interaction term in the final step of the multivariable regression models. When the interaction was statistically significant (*p* < 0.05), we performed a stratified analysis of the association between the predictor and curiosity, adjusting for the same covariates. Results were reported using standardized regression coefficients (β). In our stratified analyses, we also calculated mean parent-reported curiosity for each level of neighborhood safety (unsafe, fairly safe, very safe). *Post hoc* analyses were conducted to examine pairwise differences in mean-level curiosity between and across neighborhood safety and SES groups. Significant pairwise differences characterized by *p* < 0.05 are indicated in Figure 1. Because of the complex sample design, sample weights and the Jackknife method (Wicklin, 2010) were used to account for stratification, clustering and unit non-response, thereby allowing the weighted results to be generalized to the population of U.S. children born in 2001. Per the ECLS-B Codebook, the use of sample weights addresses attrition across waves. The appropriate sample weight was chosen based on the latest timepoint of data collection for the variables in the analytic model, thus accounting for attrition/missing data from earlier timespoints (Snow et al., 2009). In accord with the NCES requirements for ECLS-B data use, reported numbers were rounded to the nearest 50.

## Results

### Sample characteristics and neighborhood safety characteristics

At the kindergarten timepoint, 4,750 children had data on early childhood curiosity, neighborhood safety data and all covariates, which served as our analytic sample. After applying sample weights, the maternal and child characteristics were generalizable to the US population in 2001. The sample characteristics for the weighted sample, and the timespoints when each demographic variable was ascertained are shown in Table 1. Neighborhood safety characteristics at each assessment timepoint are shown in Table 2. Across the study timespoints, most families (>50%) reported living in “very safe” neighborhoods.

**TABLE 2:** Neighborhood safety (Weighted%) by assessment timepoint.

Neighborhood safety	24 months	Preschool	Kindergarten
Unsafe	7.7%	6.2%	6.9%
Fairly safe (ref)	39.3%	36.1%	33.4%
Very safe	53.0%	57.7%	59.6%

Source: U.S. Department of Education, National Center for Education Statistics, Early Childhood Longitudinal Study, Birth Cohort. Selected years 2001–2007.

To address issues of attrition, we compared demographic characteristics of children who were included in our sample (*N* = 4,750), with children who had curiosity data at kindergarten (*N* = 6,350), but who were excluded due to missing covariates (*N* = 1,600). Compared to children who were excluded due to missing data (*N* = 1,600), children included in our analytic sample were more likely to have White race/ethnicity, be enrolled in center-based preschool, have parents who were married, have higher mental BSF T-scores at 24 months, have higher quality home environments, and have higher socioeconomic status (SES).

### Main effects model: Associations of neighborhood safety, parenting quality, home environment, enrollment in center-based preschool, SES and early childhood curiosity

In adjusted models, we found evidence that *proximal* (e.g., parenting quality, quality of the home environment), *distal* (e.g., level of neighborhood safety) and *macro-level* contexts (e.g., SES) were each independently associated with parent-reported curiosity at kindergarten. Regarding the most proximal experiences in the child’s home environment, experiencing more positive parenting (β = 0.10, *p* < 0.001) and a higher quality home environment (β = 0.05, *p* = 0.02) were associated with higher curiosity at kindergarten. Enrollment in center-based preschool was not associated with higher parent-reported curiosity (β = 0.02, *p* = 0.42). Greater neighborhood safety was also associated greater child curiosity at kindergarten (*p* = 0.01), but only for children living in “very safe neighborhoods. In our main effects model, children who lived in “very safe” neighborhoods had higher levels of parent-reported curiosity at kindergarten, compared to children who lived in “fairly safe” (ref) neighborhoods, (β = 0.07, *p* = 0.002). We found no differences in parent-reported curiosity between children who lived in “fairly safe” (ref) versus “unsafe” neighborhoods (β = 0.01, *p* = 0.42). Regarding macro-level contexts, we found an association between higher SES and higher parent-reported curiosity at kindergarten (β = 0.08, *p* = 0.003) (Table 3).

**TABLE 3:** Adjusted associations of neighborhood safety, parenting quality, home environment, and SES on curiosity (main effects, step 1).

Step 1–Main effects	β (SE)	*P*
**Neighborhood safety**		
Unsafe	0.01 (0.08)	0.42
**Fairly safe (REF)**		
Very safe	0.07 (0.04)	0.002
Parenting quality (sensitive, stimulating, positive)	0.10 (0.03)	<0.001
Home environment quality	0.05 (0.02)	0.02
Attendance at center-based preschool	0.02 (0.04)	0.42
Socioeconomic status	0.08 (0.03)	0.003
Child sustained attention	−0.03 (0.02)	0.21
Maternal age	−0.07 (0.003)	<0.001
**Race/Ethnicity**		
Other	0.004 (0.09)	0.76
Asian	−0.02 (0.06)	0.03
Hispanic	0.04 (0.06)	0.13
Black/Non-Hispanic	0.001 (0.05)	0.92
**White (Ref)**		
24-month cognitive development (Bayley-SFR)	0.17 (0.002)	<0.001
Child’s sex (female)	0.07 (0.03)	<0.001

β coefficients are standardized betas.

Source: U.S. Department of Education, National Center for Education Statistics, Early Childhood Longitudinal Study, Birth Cohort. Selected years 2001–2007.

### Test of moderation: Examining whether the association between neighborhood safety and early childhood curiosity is moderated by parenting quality, home environment, center-based preschool attendance, and SES

We tested four moderation models to examine whether the association between neighborhood safety and early childhood curiosity was moderated by factors in the *proximal environment* [(1) parenting, (2) home environment quality, (3) enrollment in center-based preschool]; and the *macro environment* [(4) SES]. The association between neighborhood safety and early childhood curiosity was not moderated by parenting quality (*p* = 0.50), the quality of the home environment (*p* = 0.42), or by enrollment in center-based preschool (*p* = 0.16). We did find evidence that the association between neighborhood safety and early childhood curiosity was moderated by SES (*p* = 0.008).

### Stratified models: Examining the association between neighborhood safety and early childhood curiosity, stratified by SES tertiles

To facilitate a more nuanced examination of the association between the degree of family resource and early childhood curiosity across varying levels of neighborhood safety, we stratified by SES tertiles [lower-resourced (lowest SES tertile); middle-resourced (middle SES tertile); higher-resourced (highest SES tertile)]. This allowed us to examine how the association between neighborhood safety and early childhood curiosity varied between lower-, middle-, and higher-resourced families.

For children from *lower-resourced* environments (lowest SES tertile), we found a significant association between the level of neighborhood safety and kindergarten curiosity (*p* = 0.03), but only for lower-resourced children living in “very safe” neighborhoods. Lower-resourced children living in “very safe” neighborhoods demonstrated higher kindergarten curiosity compared with lower-resourced children who lived in “fairly safe” neighborhoods (β = 0.10, *p* = 0.009), but we found no differences in curiosity between lower-resourced children living in “unsafe” neighborhoods compared to lower-resourced children who lived in “fairly safe” neighborhoods (β = 0.03, *p* = 0.37). For children from *middle-resourced* environments (middle SES tertile), the level of neighborhood safety was also associated with kindergarten curiosity (*p* = 0.045). Similar to children who lived in lower-resourced environments, middle-resourced children living in “very safe” neighborhoods demonstrated higher kindergarten curiosity compared with middle-resourced children who lived in “fairly safe” neighborhoods (β = 0.10, *p* = 0.01). We found no differences in curiosity between middle-resourced children living in “unsafe” neighborhoods compared to middle-resourced children who lived in “fairly safe” neighborhoods (β = 0.02, *p* = 0.45). For children from *higher-resourced* environments (highest SES tertile), the level of neighborhood safety was not associated with kindergarten curiosity (*p* = 0.99). Compared to higher-resourced children who lived in “fairly safe” neighborhoods, neither living in an “unsafe” neighborhood, nor living in a “very safe” neighborhood was associated with kindergarten curiosity (β_unsafe_ = 0.001, *p* = 0.98; β_very safe_ = 0.005, *p* = 0.89) (Table 4).

**TABLE 4:** Adjusted associations of neighborhood safety, parenting, quality of the home environment and curiosity at kindergarten, stratified by socioeconomic status (SES) tertiles.

	Lower-	Middle-	Higher-
	**Resourced**	**Resourced**	**Resourced**
Step 2 results (with interaction, stratified by SES tertiles)	β (SE)	β (SE)	β (SE)
**Neighborhood safety**			
Unsafe	0.03 (0.11)	0.02 (0.17)	0.001 (0.31)
Fairly safe (ref)	–		–
Very safe	0.10 (0.07)**	0.10 (0.07)*	0.005 (0.07)
Quality of home environment	0.06 (0.03)	0.08 (0.03)*	−0.01 (0.04)
Parenting quality (Sensitive/Stimulating/Positive regard)	0.10 (0.04)**	0.09 (0.04)*	0.07 (0.05)
Child sustained attention	0.03 (0.04)	−0.06 (0.04)	−0.07 (0.03)
Cognitive development (24-months)	0.16 (0.004)***	0.15 (0.004)**	0.22 (0.003)***
**Race/Ethnicity**			
Other	−0.01 (0.16)	0.03 (0.13)	−0.04 (0.19)
Asian	0.006 (0.17)	−0.02 (0.09)	−0.05 (0.07)*
Hispanic	0.002 (0.09)	0.07 (0.10)	0.06 (0.11)
Black/Non-Hispanic	−0.02 (0.07)	0.01 (0.09)	0.03 (0.09)
White (reference)	–	–	–
Mother age	−0.04 (0.005)	−0.05 (0.005)	−0.09 (0.006)*
Child sex (female)	0.03 (0.07)	0.04 (0.07)	0.14 (0.06)***

Covariates included: child sustained attention, 24-month cognitive development, maternal race/ethnicity; maternal age; child sex. β coefficients are standardized betas. *p < 0.05; **p < 0.01; ***p < 0.001.

Source: U.S. Department of Education, National Center for Education Statistics, Early Childhood Longitudinal Study, Birth Cohort. Selected years 2001–2007.

We then examined mean levels of curiosity for lower-resourced, middle-resourced, and higher-resourced children, by level of neighborhood safety (Figure 1). We found differences for mean -level curiosity *between* neighborhood safety categories (i.e., between fairly safe and very safe neighborhoods), and *within* neighborhood safety categories (i.e., between lower-, middle-, and higher-resourced families living in fairly safe neighborhoods).

### Differences in mean curiosity between neighborhood safety categories

We found that mean-level curiosity varied between children living in fairly safe versus very-safe neighborhoods, but only for children who were lower- or -middle resourced. Children from *lower-resourced* environments demonstrated lower mean-level curiosity if they lived in fairly safe versus very-safe environments [*M*_fairly safe_ = −0.19 versus *M*_very safe_ = 0.03 (*p* = 0.003)]. Similarly, children from *middle-resourced* environments demonstrated lower mean-level curiosity if they lived in fairly safe versus very-safe environments [*M*_fairly safe_ = −0.04 versus *M*_very safe_ = 0.13 (*p* = 0.02)]. There were no differences between mean-level curiosity for lower-resourced or middle-resourced children who lived in “unsafe” versus “fairly safe” neighborhoods (*p*_lower_ = 0.46 and *p*_middle_ = 0.36, respectively). For children from higher-resourced environments, mean level curiosity did not vary across neighborhood safety categories (all *p* > 0.95). Of note, lower-resourced children demonstrated curiosity *below the mean* if they lived in “unsafe” and “fairly safe” neighborhoods, but demonstrated curiosity *above the mean* if they lived in a “very safe” neighborhood, with curiosity levels commensurate with children who were from middle-resourced or higher- resourced environments.

### Differences in mean curiosity within neighborhood safety categories

For children living in “fairly safe” neighborhoods, mean-level curiosity varied by SES tertile (i.e., between lower-resourced, middle-resourced, higher resourced environments.) We found significant differences in mean curiosity between children from *lower-resourced* and *higher-resourced* environments [*M*_lower_ = −0.19 versus *M*_higher_ = 0.11 (*p* = 0.003)]. Similarly, we found significant pairwise differences between children from *middle-resourced* and *higher-resourced* environments [*M*_middle_ = −0.04 versus *M*_higher_ = 0.11 (*p* = 0.03)]. There were no significant pairwise differences between children from *lower-resourced* and *middle-resourced* environments [*M*_middle_ = −0.19 versus *M*_middle_ = −0.04 (*p* = 0.07)]. For children who lived in “unsafe” or “very safe” neighborhoods, mean-level curiosity did not vary across SES tertiles.

## Discussion

To our knowledge, this is the first study examining the multi-level ecological contexts associated with early childhood curiosity, including the *proximal* (microsystem) environments characterized by the quality of the early caregiver-child relationship and home environment; the *distal* (mesosystem) environments characterized by the safety of the neighborhood environment and the *macro contexts* (macrosystem) associated with poverty and socio-economic disadvantage. In adjusted, stratified models, our results demonstrated that children from lower-, and middle-resourced environments manifested higher curiosity if they experienced more positive parenting, higher quality home environments, and if they lived in neighborhoods that were “very safe.” While we did not find an association between living in “unsafe neighborhoods” and early childhood curiosity across SES categories (lower-, middle-, higher-resourced), we found an association between living in a “very safe” neighborhood (compared with living in a “fairly safe” neighborhood) and higher kindergarten curiosity for lower- and middle-resourced families. This suggests that for less-resourced families, living in a “very safe” neighborhood may be an important, potentially modifiable context to promote a social-emotional process (i.e., curiosity) associated with early academic achievement.

### Neighborhood safety and early childhood curiosity

We consider our results in light of the characteristics that underly early childhood curiosity, and consider implications for interventions focused on neighborhood quality and safety. Curiosity is characterized by intrinsic motivation [i.e., *the drive to understand what one does not know* (Hall and Smith, 1903)] and an exploratory drive (e.g., *an exploratory drive to seek novelty* (Berlyne, 1954). In young children, curiosity is largely manifest through expressions of play and exploration (Schulz and Bonawitz, 2007; Cook et al., 2011), and is believed to be influenced by environmental characteristics that either promote or restrict the expression of that exploratory drive. Because a fundamental aspect of curiosity is the ability to engage in exploration, we considered that neighborhoods that were more *unsafe* might be associated with *lower* child exploration (and *lower* levels of *curiosity*), and conversely, that neighborhoods that were *more safe* might be associated with *higher* child exploration (and *higher* levels of *curiosity*).

We found partial support for our hypothesis, but only for children from select *lower-, and middle-resourced* environments. While prior research has suggested that lower neighborhood safety and greater neighborhood poverty are associated with maladaptive child social-emotional outcomes including lower effortful control (Tomlinson et al., 2020), and greater amygdala reactivity to neutral faces (Hyde et al., 2020), and while there is some evidence suggesting that parents who live in unsafe neighborhoods discourage outdoor play and exploration (Dias and Whitaker, 2013), we did not find and association between living in an unsafe neighborhood and lower expressions of curiosity at kindergarten. This may be partially explained by the fact that other, more proximal contexts (e.g., parenting quality and quality of the home environment, described in further detail below) may (independently) support the development of early childhood curiosity, irrespective of the safety of the neighborhood.

While we found no differences in mean-level curiosity between lower-resourced children living in “fairly safe” versus “unsafe” neighborhoods, we observed that lower-resourced children living in “very safe” neighborhoods manifested higher curiosity compared to lower-resourced children who lived in “fairly safe” neighborhoods. Notably, compared to children from higher-resourced environments, children from lower-resourced environments manifested curiosity below the mean if they lived in “fairly safe” environments, but those gaps in early childhood curiosity between lower- and higher-resourced children were eliminated if lower-resourced children lived in “very safe” neighborhoods. Our findings suggest that for lower-income children, living in a “very safe” neighborhood is a potentially modifiable factor which can essentially close the “curiosity gap” observed when lower-resourced children lived in less-safe environments.

Our findings which indicated no differences in curiosity between lower-resourced children living in “unsafe” versus “fairly safe” environments was surprising, for which we offer a possible explanation. We consider that for lower-income children, higher curiosity may be related to a child’s ability to play and explore in their neighborhood. For lower-resourced families (i.e., lowest SES tertile), we theorize that this ability to play in their neighborhood is possible only if parents consider their neighborhood to be “very safe.” For lower-income families, environments which are considered “unsafe” or even “fairly safe” may still feel “too dangerous” to allow for play and exploration in the neighborhood. Relatedly, it is notable that for higher-resourced families, we found no differences in mean curiosity across neighborhoods (“unsafe,” “fairly safe,” “very safe”). One possible explanation is that for higher resourced families, children’s curiosity is also cultivated through involvement in other activities, rather than predominantly through play and exploration in their neighborhood. There is some evidence to suggest that irrespective of neighborhood characteristics, more resourced families are more likely to enroll their children in recreational activities to foster child engagement, compared to less-resourced families (Galaviz et al., 2016). Taken together, this suggests that while higher-SES families may have resources to access supplemental activities which may foster curiosity, lower-SES families may have less access to these opportunities, which makes the context of neighborhood safety, and the possibilities for play and exploration within their neighborhood, an especially relevant context for the promotion of curiosity.

### Neighborhood spaces as a modifiable ecological context to foster play and curiosity

As children grow, they spend more time in the neighborhood, which becomes a salient ecological context for play, exploration and learning. Some neighborhood characteristics, described by the quality of the built environment (Pearl et al., 2001; Davison and Lawson, 2006) appear to be especially salient to child development, and include the physical properties of the neighborhood such as the presence of outdoor play spaces, and the ability to engage in them safely. There is research to support the benefits of designing everyday neighborhood spaces like bus stops, libraries and supermarkets in ways that are promotive of play, exploration and learning (Hassinger-Das et al., 2018, 2020, 2021; Schlesinger and Hirsh-Pasek, 2019).

Playful Learning Landscapes (PLL), for example, evolved as a collaboration between researchers at the Playful Learning Landscape Action Network and the Brookings Institute, with the aim of transforming spaces where families wait (e.g., bus stops, supermarkets and laundromats) into hubs that could promote academic and social enrichment, (Hassinger-Das et al., 2018, 2021). Urban Thinkscape, one example of a playful learning landscapes evolved as a collaboration between local community leaders in Philadelphia and child development researchers, with the aim of “marrying” architectural design with the science of learning by creating a playground installation to foster caregiver-child conversations around topics foundational to school readiness. The location for Urban Thinkscape was selected based on 3 criteria: (1) >50% residents living below the poverty line; (2) geographic areas in need of accessible play spaces; and (3) presence of community organizations. Working with community members, the researchers melded neighborhood values with the science of how and what children learn and literally worked these into the built environment. For example, a lot on which Martin Luther King delivered one of the Freedom March speeches was chosen as the designated space. Large rotating puzzles along with installations sparking inhibition control and shape identification dotted the new space where people waited to board a city bus. Results demonstrated that local spaces could be crafted into socially interactive spaces designed to scaffold and support children’s early learning experiences in language, literacy and STEM skills (Hassinger-Das et al., 2020).

The mechanism by which projects such as “Playful Learning Landscapes” and “Urban Thinkscape” is thought to promote early learning is by enhancing opportunities for social interaction and conversational exchanges between parents and young children (Schlesinger and Hirsh-Pasek, 2019). Prior research demonstrates that that socioeconomically disadvantaged children preferentially benefit from greater child-directed speech and conversational exchanges (Zimmerman et al., 2009; Vernon-Feagans et al., 2013; Hirsh-Pasek et al., 2015a; Pace et al., 2017), and that children learn best in environments that are *interactive*, which encourage *turn-taking*, *active engagement, dialogic exchanges*, and *intrinsically motivated questions* (Zimmerman et al., 2009; Hirsh-Pasek et al., 2015a, b). There is evidence suggesting that initiatives which transform neighborhood environments into safe, playful learning spaces, promote caregiver-child interaction and interactive discourse, contributing to active and engaged child learning (Hassinger-Das et al., 2020).

We theorize that these same “built environments,” especially in disadvantaged communities, can foster *early childhood curiosity* by creating interactive opportunities to engage in conversational exchanges that are dotted with questions (Gaudreau et al., 2021). Our previous research found an association between more frequent parent conversation (during share television viewing) and higher kindergarten curiosity, with a greater magnitude of association in children from low-SES families (Shah et al., 2021). Research from Playful Learning Landscapes including Urban Thinkscape and other projects like Parkopolis (Bustamante et al., 2020); a life-sized human board game) and Fractionball (Bustamante et al., 2022); a recrafted basketball court designed to promote fraction talk) demonstrate that transforming neighborhood spaces into areas which prioritize caregiver-child interactions, facilitate language-learning opportunities which are promotive of school readiness (Hassinger-Das et al., 2018) and question asking–a behavior associated with curiosity. Transforming neighborhoods into safer, more playful spaces can also lead to the promotion of early childhood *curiosity* by cultivating opportunities to engage in back and forth pedagogical exchanges, (e.g., “*Look at this!”; “What does that mean?”; How does it work?”; “Why does it do that?*”).

### Proximal contexts to promote early childhood curiosity: The role of positive parenting and quality home environments

In addition to the distal ecological context of the safety of the neighborhood environment, our results also support the importance of the quality of the proximal caregiving environment for the expression of early childhood curiosity. For children from lower and middle-resourced environments, they demonstrated higher curiosity if they experienced more positive parenting (manifest as more sensitive, stimulating, and attuned parenting), and, for middle-resourced children, if they experienced higher quality home environments (manifest by higher safety, supervision, and stimulation). Curiosity is manifest by exploratory behavior that is intrinsically motivated (Kashdan and Silvia, 2009). The expression of curiosity is enhanced when individuals are allowed to engage in activities that are align with their idiosyncratic interests (Black and Deci, 2000), however, for young children, they require the support and scaffolding of their caregivers to effectively engage with their environment (Sameroff, 1975). Attachment theory considers that children who experience more sensitive and scaffolding parenting in infancy are more likely to have secure attachment, manifest by greater secure-base exploration (Bowlby, 1969; Ainsworth et al., 1978). Sensitive, scaffolding parenting has been shown to be promotive of numerous adaptive social-emotional processes including self-regulation (Calkins, 2011), effortful control (Regueiro et al., 2020), and executive function (Valcan et al., 2018). Our results similarly suggest that sensitive, scaffolding, positive parenting is also promotive of early childhood curiosity, with a greater magnitude of association for lower-income children.

Cumulative risk models also demonstrate how environments of socio-economic disadvantage, in combination with low-quality home environments are detrimental to young children’s social-emotional development (Watamura et al., 2011). Disadvantaged home environments characterized by low environmental quality (i.e., lower home learning experiences) and less positive parenting practices have been linked with less optimal social-emotional outcomes (Foster et al., 2005), including lower self-regulation (Raikes et al., 2007). However, the converse has also been shown: for low income children, more stimulating home environments, and more sensitive, scaffolding parenting have also been associated with higher academic achievement, and lower child behavior problems (Longo et al., 2017). Our findings, demonstrating an association between more positive parenting, higher quality home environments and higher curiosity at kindergarten similarly align with a growing body of evidence highlighting the importance of the quality of early caregiving to foster more adaptive social-emotional outcomes in lower-income children.

We consider our results in consideration of the factors that are believed to foster curiosity. Young children have an intrinsic desire to explore and make sense of the world around them (Piaget, 1926), and environments which foster exploration, discovery and pedagogical inquiry are thought to promote the expression of curiosity (Jirout, 2020). In school-age children, parents who ask questions that align with their children’s interests, and who provide exposure to new experiences, and encouragement to seek new knowledge, have children who were rated higher in scientific curiosity in middle childhood (Gottfried et al., 2016). For *young children*, our results suggest that similar conditions are also promotive of curiosity, including *greater child-directed conversation* (e.g., the parent talks more to the child, and encourages pedagogical exchanges); *safe, stimulating home environments* (e.g., parent provides adequate supervision, offers toys for exploratory play); and *sensitive and scaffolding parenting interactions* (e.g., parent is attuned to the child’s idiosyncratic interests, and demonstrates sensitive support in dyadic interactions).

## Limitations

Our study had several strengths and limitations. Strengths include the use of a nationally representative sample which included (1) child behavioral data (from which we could derive a measure of curiosity), (2) measures of multi-level ecological contexts salient to child development and (3) data from 9 months to kindergarten. One limitation is that our curiosity factor was derived from a single parent-report behavioral measure at the kindergarten timepoint. As such, we acknowledge the potential bias and shared method variance associated with parent report measures. In addition, our measure of neighborhood safety was based on a parent-report questionnaire, and additional objective details about neighborhood characteristics including levels of poverty, violence and other community characteristics were not available. Relatedly, another limitation relates to the lack of clarity on the measurement of environmental safety among lower-, middle-, and higher-resourced families. In addition, with SES divided into tertiles, small sample sizes for some groups, (e.g., few higher-resourced families lived in “unsafe” neighborhoods), likely contributed to a lack of statistical significance in pairwise comparisons (e.g., between lower-resourced. middle-resourced, and higher-resourced children in “unsafe” environments). Relatedly, while the magnitude of association between neighborhood safety and curiosity across SES categories was significant, these associations were on the lower side of the effect size. However, given that our results are drawn from a nationally representative sample, at a population level, even small effect sizes are impactful for population-making decisions. In addition, we acknowledge the limitation that some participants were not included in the final sample due to attrition or missing data. Finally, while the ECLS-B is a rich dataset and among the few longitudinal cohorts from the United States, the dataset did not include outcomes beyond the kindergarten timepoint. Future research should consider the association between early childhood curiosity and outcomes throughout the childhood lifespan, (Kashdan et al., 2013b), and should examine the pathways through which cultivation of neighborhood spaces may foster curiosity and help mitigate the poverty achievement gap (Grogan-Kaylor and Woolley, 2010). Despite these limitations, we believe that our results have some important implications for caregivers, pediatricians and policymakers.

## Implications and conclusion

Building on our previous work which identified an association between higher early childhood curiosity and higher academic achievement, with a greater magnitude of effect in low-income children (Shah et al., 2018), we identified several ecological contexts in early childhood (i.e., the proximal contexts of the home environment and parenting quality and the distal context of neighborhood safety) associated with higher curiosity in under-resourced children. Our results identify several areas that can serve as potential targets of intervention to foster early childhood curiosity. Environments which support scaffolded exploration, questioning, and discovery has been shown to promote early learning (Hirsh-Pasek et al., 2015b). Thus, interventions which target parent scaffolding and promote opportunities for conversational exchanges, may be similarly beneficial for the cultivation of curiosity, especially in lower-resourced children (Hirsh-Pasek et al., 2015a). We also found that higher neighborhood safety (i.e., living in a “very safe” neighborhood) was associated with higher early childhood curiosity in lower-income children. Novel community-based partnerships in under-resourced communities have demonstrated that transforming neighborhood spaces into areas which prioritize caregiver-child interactions fosters language-learning opportunities which are promotive of early learning (Hassinger-Das et al., 2018). We believe that such interventions which transform neighborhoods into safer, more playful spaces (especially in lower-resourced environments), may also lead to the promotion of early childhood curiosity. Future research should consider the mechanisms and pathways by which safe neighborhoods foster early childhood curiosity, especially for families with socioeconomic disadvantage.

## Data availability statement

Publicly available datasets were analyzed in this study. This data can be found here: Data can be accessed after entering into a data-use agreement with the Institute of Educational Statistics, National Center for Educational Statistics.

## Author contributions

All authors have participated in the concept, design, analysis or interpretation of data, and have assisted with the drafting or revising of the manuscript and responsible for the reported research, and have approved the manuscript as submitted.

## Appendix

**TABLE A1:** Derived curiosity factor from the ECLS-B.

Question Items in Derived Curiosity Factor	Loading Coefficient
Likes to try new things	0.71
Shows imagination in work and play	0.58
Shows eagerness to learn new things	0.59
Easily adjusts to a new situation	0.56

Cronbach’s alpha: α = 0.70.

Adjusted GFI = 0.91.

Standardized RMR (SRMR) = 0.047.

Source: U.S. Department of Education, National Center for Education Statistics, Early Childhood Longitudinal Study, Birth Cohort. Selected years 2001–2007.
